# Acoustic effects complement visual displays of Great Bowerbird bowers

**DOI:** 10.1093/beheco/arae070

**Published:** 2024-09-07

**Authors:** John A Endler, Selina Meehan, Aida Rodrigues, Vicki Hallett

**Affiliations:** Centre for Integrative Ecology, School of Life and Environmental Sciences, Deakin University, Waurn Ponds, Victoria 3216, Australia; Zoology and Ecology, College of Science and Engineering, James Cook University, Smithfield, Cairns, Queensland 4878, Australia; Centre for Integrative Ecology, School of Life and Environmental Sciences, Deakin University, Waurn Ponds, Victoria 3216, Australia; Centre for Integrative Ecology, School of Life and Environmental Sciences, Deakin University, Waurn Ponds, Victoria 3216, Australia; Hallett Environmental Sounds, 18 Chenin Mews, Waurn Ponds, Victoria 3216, Australia

**Keywords:** Bowerbirds, bower acoustics, bower design, constructed signals, *Ptilonorhynchus nuchalis*, multimodal signals, sexual display, sexual selection

## Abstract

Sexual selection can result in extreme development of multimodal mate-attracting traits, including complex constructions. Male Great Bowerbirds build bowers for attracting females. Bowers contain a thatched twig tunnel (avenue) opening onto 2 courts covered with decorations. Males displaying on a court are seen by a female from within the avenue. She sees and hears displays through the avenue entrance but can only see the male’s head and objects in his bill as it passes repeatedly across the entrance. Because the bower may affect the auditory as well as the visual parts of the multimodal male display we investigated bower acoustic properties by playing standard sounds from multiple court positions, recording the resulting sounds at the female’s head position within the avenue. Bower geometry results in a limited zone at the avenue entrance where his vocalisations can be heard with maximum intensity; this corresponds to his typical display position. Experiments show that court decorations increase the intensity of some frequencies and reduce the intensity of others. Bower structure simultaneously affects both visual and auditory male display components and could be important in sexual selection. It is important to consider more than 1 sensory mode, especially in the context of built signaling structures.

## Teaser

Sexual selection can result in complex traits. Bowerbirds construct and decorate bowers which are used to attract and hold a female’s attention for mating while she watches from within the bower tunnel. Bowers allow a complex visual display but the structure also affects the sounds reaching the female. We showed that the structure limits the locations from which a male can transmit the loudest sounds to the female and also affects the pitch structure.

## Introduction

Sexual selection can generate complex traits used in mate choice and can arise from several different sexual selection processes (Kokko et al. 2003). Many sexual signals have components transmitted in several sensory modes, and multimodal signaling is often associated with improved mating success (Mitoyen et al. 2019). Some species modify the environment or make structures in order to produce stronger visual or auditory signals, examples include Bowerbirds (Frith and Frith 2004), Cock-of-the-rock (Endler and Théry 1996), Leptodactylid frogs (Muñoz and Penna 2016), Gobies (Lugli 2012), Tree crickets (Forrest 1991), and Mole crickets (Daws et al. 1996), but these studies only consider a single sensory mode. Here we investigate how bower structure can be used in simultaneous multimodal signaling in Bowerbirds.

**Fig. 1.**
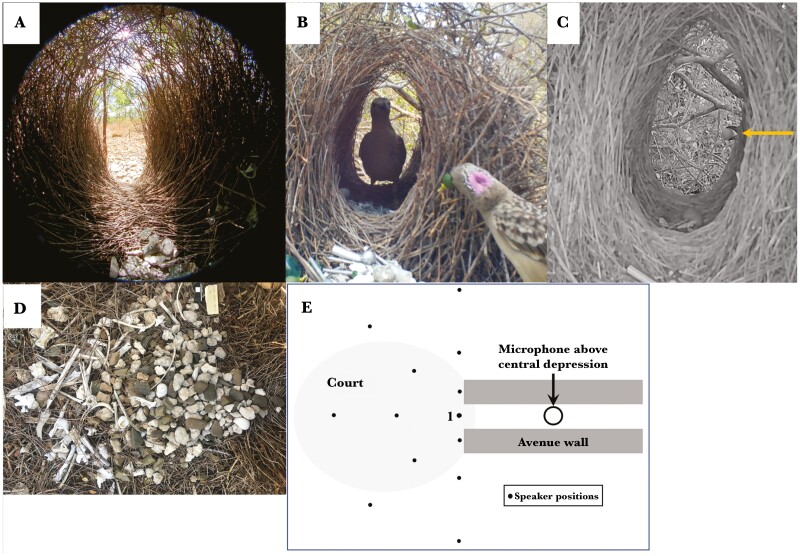
Bower geometry. A) Hemispherical photo (360° solid angle) taken at the female’s head position inside the avenue, note ornaments in central depression below her head position and pebbles on the court outside the far avenue entrance. B) Photo of female in avenue and male displaying a fruit in his bill at the avenue entrance. His nuchal crest will be expanded further when he turns his head around to show it to her. Note ornaments at avenue entrance. C) Photograph of male bill holding a fruit at the opposite entrance of the avenue (note arrow) when displaying without a female. D) Court photo with end of avenue at right; the other court is at the opposite end of the avenue and is usually smaller and darker. E) Speaker positions (dots) relative to main court and avenue. Position 1 is in front of the avenue entrance.

Male Bowerbirds construct and decorate a bower using locally available objects and use it to attract and stimulate females to mate (Frith and Frith 2004). Male Great Bowerbirds (*Ptilonorhynchus* = *Chlamydera nuchalis*) build a bower consisting of a 60–100 cm thatched stick tunnel (the avenue) opening onto 2 courts (Fig. 1). Courts and avenues are constructed by males and covered with stones, bones, and bleached snail shells (gesso, see Endler et al. 2010), and a few colored objects (mostly green and red) are placed on the sides of the courts (Endler et al. 2014), close to the entrance, with 1 court larger and often more well-lit (the main court). Some objects are man-made such as glass, metal, and plastic (Endler et al. 2014). Objects are also placed in a depression in the middle of the avenue floor (Fig. 1A). Male courtship displays take place on a court near the entrance while the female watches from inside the avenue (Frith and Frith 2004), Fig. 1B. She sees a complex visual display but can only see the male’s head, with or without colored objects in his bill, whenever it passes within her field of view through the avenue entrance (Endler et al. 2014), Fig. 1C.

**Fig. 2.**
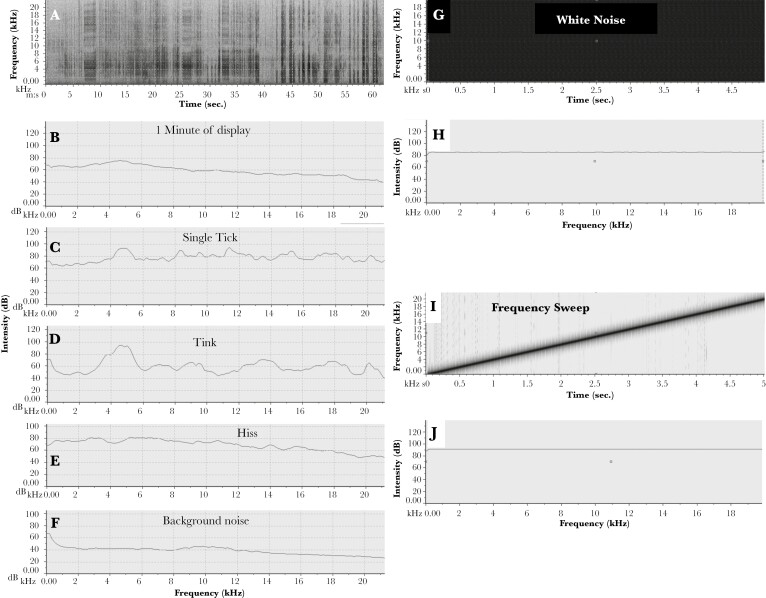
Justification for design of experimental sound. (A–F) natural and (G–J) experimental sounds. A) spectrograph of characteristic sounds made by a great bowerbird male displaying on a court to a female inside the bower (scale 0–21 kHz by 0–60 s.). B–F) Sound spectra of sounds isolated from the recording (0–100 or 120 dB by 0–21 kHz); spectra are averages over the sound’s time interval using RavenPro. The background noise F) was extracted from a quiet period in the same recording. Sounds made during displays are 20–40 db above the background noise. G) white noise playback spectrograph, a mixture of many frequencies (0–20 kHz by 5 s). H) sound spectrum of (G) (0–120 dB by 0–20 kHz). I) Frequency sweep playback spectrograph. J) sound spectrum of sweep (same scale as H). Playback spectra are flat, allowing simple estimates of sound properties of the bowers when recorded in the avenue at the female’s head position. The spectral shapes are more important than the details of the axes. Axes ranges (x, y) are: A: 0–60 s, 0– 22 kHz. B–F: 0–20 kHz, 0–120 dB (B–E) and 0–100 db (F). G: 0–5 s, 0–20 kHz. H: 0–20 kHz, 0–130 dB; I: 0–5 s, 0–21 kHz. J: 0–20 kHz, 0–130 db.

The visual appearance of the bower plays a very important part in courtship and mating success, as does male display behavior (Frith and Frith 2004; Endler et al. 2005; Endler and Day 2006; Endler et al. 2010; Kelley and Endler 2012a, 2012b; Endler et al. 2014). The courtship sequence is as follows (Frith and Frith 2004): When a female arrives near a bower the male will vocalize and then fly down to 1 court. If a female goes inside the bower avenue, the male starts his visual display on the court, consisting of picking up colored objects and sticks out of sight of the female in the avenue, waving them and alternatively his turquoise nuchal crest, across the avenue entrance where the female can see them briefly, until he throws them out of her sight next to the entrance (Endler et al. 2014; Kelley and Endler 2021a). Simultaneously he gives off broad-band vocalizations consisting of hisses and ticks (Fig. 2; Okida et al. 2010). If the female stays in the avenue he runs around to the opposite court, enters the avenue, and copulates with her, see online video.

Males produce a continuous vocalization during the display as they move their head in and out of the female’s field of view and walk on the bower court (Frith and Frith 2004; Endler et al. 2014; Endler pers. Obs; Okida et al. 2010), see Fig. 2 and the online video. Males often push the avenue wall (where the male’s head is in Fig. 1B), making a rustling crackling sound. When he walks on the court, or throws an object, the empty snail shells may make a “tink” sound and bones and stones will make a “clack” sound. However, his main sound is his vocalization, which occurs throughout his visual displays. Given the tunnel-like avenue and the male only spending part of his time directly in front of the avenue entrance during displays, the bower structure could affect how the male vocalizations are received by the female during his display. Moreover, the hard objects in the bower are more efficient sound reflectors than the rest of the environment near the bowers (Dusenbery 1992).

It is intuitively obvious that geometry affects the directionality and sound intensity of signals but this has not been tested before except in Tree crickets (Forrest 1991) and species such as Mole crickets (Daws et al. 1996) and other taxa using burrows or holes. In these species, males call in a structure and females are outside. Unlike other species which use constructions for mating, male Bowerbirds call outside the avenue and female Bowerbirds listen within the avenue; in other species, males call inside the structure or burrow. In addition, the sound frequency response of bowers is unknown.

Here we investigate experimentally how the bower structure and bower objects affect the relative sound amplitude and frequency spectrum arriving in the avenue at the female’s head height. We have 2 hypotheses about the sound arriving at the female’s head position during the display which arise from direction- and sound-frequency-specific reflection (Dusenbery 1992): (h1): the tunnel shape of the bower avenue affects the geometry of sound intensity, hence the best place for the male to display; and (h2) the gesso (hard objects) on the courts and avenue floor affects the sound spectral shape and relative intensity inside the avenue because hard objects affect the sound reflection spectrum.

## Methodology

### Field site

We measured undisturbed naturally occurring bowers of Great bowerbirds (*Ptilonorhynchus* (=*Chlamydera*) *nuchalis*) at Dreghorn station (20.258 S 147.738 E; Doerr 2009; Endler et al. 2014). Bowers are about 550 m apart and under shrubs in eucalyptus woodland, which is typical for the species’ geographical range. We measured 4 bowers in 2015, 5 bowers and 3 controls in 2021, and 10 bowers and 6 controls in 2023, all in the active season, late September to early October. Bowers were chosen if active, as shown by the presence of fruits and other colored objects on the bower (Endler et al. 2014). Control sites had the same speaker and microphone geometry as bowers, but were done in areas with no bower or shrubs and were less than 100 m from the nearest bower.

### Standard playback sound

Bowerbird males make a characteristic set of sounds (Fig. 2A–F, see also Okida et al. 2010) at the bower court when actively displaying to a female in the bower avenue (Fig. 1B). The simultaneous visual and vocal display does not guarantee a mating although longer bouts are more likely to be successful (Endler et al. 2014). We used typical recorded Great Bowerbird sounds to create a standard playback sound with parameters similar to natural vocalizations.

In order to obtain parameters for the test sounds we recorded the display sounds at a bower with a Sennheiser MKE2 Gold Omni Lavalier Microphone and a Zoom H5 recorder placed next to the avenue at the same time as a motion-activated video camera recorded displays (as in Endler et al. 2014), (Fig. 2A–E). Males do not tolerate a microphone or other foreign object inside their avenue when courting a female; they immediately remove and peck at them, hence the microphone placement on the avenue side rather than inside. Background noise was 20 to 40 dB lower than the Bowerbird vocalizations, calculated within any signal (Fig. 2F). We also examined multiple recordings of display sounds by ourselves using different sets of equipment (16 bits/sample at 44.1 kHz, including the zoom recorder) and by others (Okida et al. 2010; Xeno-Canto Foundation 2022). All recordings showed qualitatively very similar sound spectra. We were not interested in absolute intensity, only spectral shape for the purposes of designing playback stimuli.

Figure 2A–F and Supplementary Figure S1 show the sound properties of all sounds and each kind of sound recorded with the MKE2 microphone, extracted and analyzed with RavenPro 1.4 software. There are 2 short components, ticks, and tinks. Ticks are bursts of almost white noise repeated through much of the display whilst tinks are single almost bell-like chirps and are much less frequent. Okida et al. (2010) recorded the western subspecies (*P. n. nuchalis*) ticks and found it consisted of a series of harmonics; these are barely visible in our recordings of the eastern subspecies (*P. n. orientalis*). Tinks could result from walking over empty snail shells, dropping shells during displays, or sound mimicry. For example, we have heard tinks in a few video recordings where the male walks over a court with empty snail shells. The hiss is longer (Supplementary Fig. S1) and is similar to a rectangle in the spectrograph, sometimes with the bands found by Okida et al. (2010). These sounds have fairly flat spectra (Fig. 2B–F). The broadband nearly flat spectra are the basis of our standard sound parameters.

To provide a standard sound for playbacks at the bowers and controls we constructed two different kinds of playback sounds using MATLAB 2021b with the same frequency range found in natural auditory displays, but flatter (Fig. 2 H, J compared to B–F) because we explicitly wanted to investigate the direct effects of the bower on the standard sound spectrum arriving inside the bower avenue. Using natural sound recordings (which are not flat) would have made examining the frequency response of the bower complex and with the added problem of noise within and variation among natural recordings (Fig. 2A). We therefore used a uniform mixture of frequencies (white noise) in 2015 (Fig. 2G, H) and a frequency sweep (Fig. 2I, J) in 2021 and 2023. The purpose was to play known replicable sounds from various positions on the main court and record what arrives at the female’s head position in the avenue (Fig. 1B). The main court is the court where the male displays most often; it has slightly more light and has more gesso than the other court, as seen in multiple display video recordings (Kelley and Endler 2012a; Endler et al. 2014).

### Microphone configurations

Females listen with their heads over the avenue central depression and face the court where the male displays. Consequently most sound would come through the entrance where the male is displaying, but sound can also diffuse around to the other avenue entrance to the female’s back as well as through the avenue roof. Ambient noise can come from all directions, although it is 20 to 40 dB lower than the vocalizations (Fig. 2). To test the effects of sound from the main entrance and from elsewhere we used 2 different experimental configurations: (1) *directional* (2015 and 2021), to differentially record sound coming through the main avenue entrance to the female head position, and (2) *omnidirectional* (2023), to record sound coming from all directions at the female head position. In both cases, the microphone was positioned with its tip (directional) or center (omnidirectional) over the central avenue depression at the typical female head height. Playback positions (Fig. 1E) were designed to explore the effects of distance and angle to the avenue entrance while keeping the receiving position constant in the center of a female’s head excursions. The 2 configurations were explicitly designed to account for sound diffusing to the female from all directions. Directional was set up to minimize ambient noise from the back avenue entrance and concentrate on sound coming through the male side avenue entrance whereas omnidirectional measured all sound, regardless whether it came from the avenue entrance, overhead through the ceiling, and the back entrance. The difference is a measure of the directionality caused by the bower avenue.

### Experiments

We used 4 experiments to test the 2 hypotheses about structure affecting sound during the male’s display (h1) avenue geometry and (h2) gesso (stones, bones, and bleached empty snail shells). Experiments were carried out in 2015, 2021, and 2023 after finding active bowers.

#### Experiment 1 (2021) Directional geometry experiment.

**Fig. 3.**
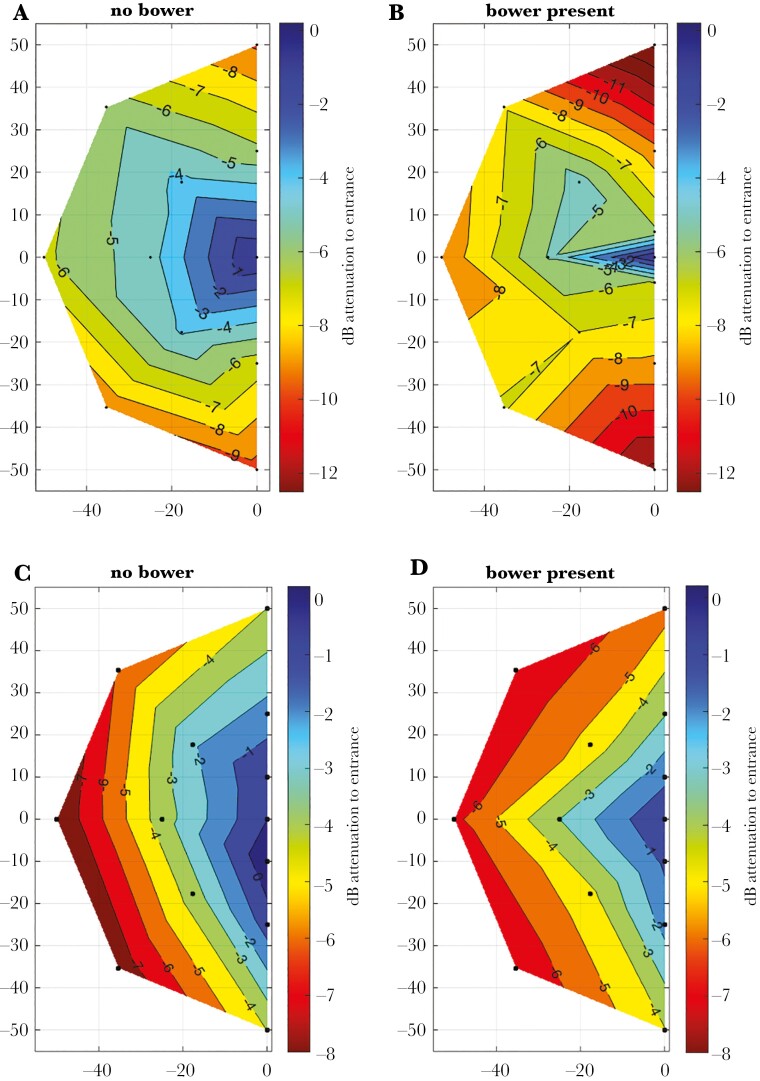
Intensity at the microphone as a function of the speaker playing a standard sound at each of the 13 positions (dots). The microphone is about 30 cm from the avenue entrance at position 1 (coordinates 30,0, not shown here). All data are relative to the intensity recorded when the speaker was at position 1 (avenue entrance) within a bower. A) directional microphone, no bower (control); B) directional microphone in avenue; C) omnidirectional microphone no bower; D) omnidirectional microphone in avenue. A–B: experiment 1, C–D experiment 2.

The sweep test sound (Fig. 2I, J) was broadcast from various positions (Fig. 1E) on the main court (Fig. 1D) using a Marantz PMD661 digital recorder resting its narrow end on the main court (Fig. 3B) with its speaker always facing towards position 1. The Marantz speaker shows less than 0.5 db change when within ±20° of perpendicular to the microphone (Supplementary Fig. S2), and our avenue entrance aiming error was less than 5°. Recordings of the played sounds were made with an AudioTechnica AT8035 directional microphone connected to a second Marantz PMD661 recorder set to record 16 bits/sample at 44.1 kHz. The input gain was set to 4.5 for clipping at a maximum −8dB. These settings were constant for all 4 experiments. The microphone was placed in the avenue facing the main court with the tip centered over the avenue’s central depression, at the approximate average position and height of the female’s head (Fig. 1C) seen in video recordings (Endler et al. 2014); about 4 cm below the avenue roof. It was held in a flexible tripod mounted just outside the opposite avenue entrance. Females usually stand with their heads over the central depression (Fig. 1B) but move their heads around during the display, and face the male avenue entrance (see online video). This experiment concentrates on sound coming through the entrance from the displaying male.

#### Experiment 2 (2023) Omnidirectional geometry experiment.

The test sound sweep (Fig. 2I, J) was played back from various positions on the main court (Fig. 1E), using a Bose Revolve II SoundLink with omnidirectional radiation. This transmits higher frequencies better than the Marantz (Supplementary Fig. S3). Although the Bose speaker is omnidirectional, for consistency we always placed it with the USB charge socket consistently facing away from the bower entrance. We saved the test sound in an iPhone12 and sent the sound to the speaker via bluetooth. We placed a matched pair of FEL Communications Clippy XLR EM272 Stereo Microphones back-to-back within a 5 cm diameter Styrofoam ball with their faces flush with the ball surface, with close to 360° solid angle reception. This is larger than a Bowerbird head but the microphone lengths set the minimum width and the ball diameter is smaller than the female head excursions whilst watching the male. We oriented the ball at female head height with its left and right microphones facing the left and right avenue walls. It was held up with two 2 mm wooden skewers embedded in a foam stand resting on the central depression. The microphone pair was connected to both channels of a Marantz PMD661 digital recorder with the same settings as in experiment 1. Both channels were converted from dB to power, added, and converted back to dB before further analysis. This configuration accounts for sound entering the avenue from the entrance, but also through the roof, through the avenue walls and the opposite entrance. The Clippy pair sound spectrum was similar to but slightly flatter than the AudioTechnica microphone (Supplementary Fig. S3A).

For both experiments 1 and 2, control recordings were also made in open areas away from a bower or vegetation with the same geometry (Fig. 1E) to estimate the geometric and acoustic effects of the speaker and microphone properties on the received spectra to compare with bowers. The microphone was placed 30 cm from simulated position 1 (Fig 1E, not shown in Fig. 3), simulating a typical distance to the avenue central depression. All recordings were relative to position 1 within a bower or control.

#### Experiment 3, Gesso progressive removal (2015).

Gesso consists of stones, bones, and bleached snail shells on the courts and often in the central depression (Endler et al. 2010, 2014). Colored court ornaments are normally outside the female’s field of view until actively picked up and displayed by the male (Endler et al. 2014), so here we concentrate on the gesso. Colored objects are soft and represent a very small fraction of a bower surface area, whereas gesso objects are hard and will be more sound reflective and spectrally different than soft objects (Dusenbery 1992). We removed different classes of gesso objects from the main court and central depression, and also filled shells with clay to affect their sound impedance. Standard white noise sounds were played from position 1 (Fig. 1E) and the 2 other positions along the avenue axis (to the left of position 1 in Fig. 1E) before (intact bower) and after manipulation. The directional microphone was in the same position as experiment 1 but here we used the white noise test sound (Fig. 2G, H). The progressive removal of different kinds of gesso allowed exploration of their relative effects of each kind of gesso on the sound frequency spectrum inside the avenue.

#### Experiment 4, Gesso removal (2023).

We removed all the gesso, leaving only the underlying twigs and soil. We used the same configuration and sound as in experiment 2, omnidirectional microphone and sound sweep. In some bowers the gesso is piled up against the avenue entrance raising position 1 above the rest of the court; when this was removed we set the speaker at the original height of position 1 (relative to the avenue floor) by resting it on some objects.

### Sound analysis

Test recordings were converted from power to frequency spectra (dB vs sound frequency), using the MATLAB 2021b functions *audioread*, *pspectrum*, and *db*. The artificial sound spectra were flatter than the Bowerbird sounds (Fig. 2) in order to explore the auditory properties of the bower avenue at each frequency rather than being a perfect mimic of natural sounds, and also to make up for the relatively weaker high-frequency response of speaker and microphone. The 2015 speaker and microphone (experiment 3) had the narrowest (least flat) spectrum, 2021 (experiment 1) flatter and more sensitive, and 2023 (experiments 2 and 4) most sensitive (Supplementary Fig. S3). To further minimize equipment spectral effects of the speaker and microphone we subtracted all spectra (frequency-by-frequency) and intensities from matching recordings of that bower or control site from position 1 (directly in front of the avenue entrance, Fig. 1E). This eliminates the need for exact intensity calibration, analogous to using a white standard in color research. To examine spectral shape independent of intensity in the generalised additive models (GAM) analyses we added the minimum value to all frequencies for a given spectrum, thus setting the minimum to 0 (instead of a negative value) and then divided by the total and multiplied by 100 to make each spectrum have the same total intensity but retaining any shape differences.

### Statistical analysis

Relative intensities were analyzed in R by GLM (generalised linear models). Standardised spectra were analyzed by GAM and results were plotted using the R (R Core Team 2023) packages mgcv (function gam, Wood 2022) and itsadug to allow for autocorrelation within spectra (plot_smooth, van Rij et al. 2022). Plots include 95% confidence limits (CL); when they do not overlap the relative intensities of the non-overlapping frequencies are significantly different. R scripts are in Supplementary Table S1. We treated bower or control locations as random effects, which also controls for varying avenue lengths, hence varying distances between position 1 and the microphone among bowers. For controls (bower absent) the distance between position 1 and the microphone was set at 30 cm.

## Results

### Geometry (experiments 1 and 2).

Figure 3 shows the relationship between angle and distance from the speaker to the avenue entrance (position 1 or location 0,0 in this figure) for sound intensity received at the microphone. The slight asymmetry in Fig. 3C is due to the left and right microphones stacked in a vertical plane, but it largely disappears in the avenue (Fig. 3D). The GAM analyses (Supplementary Tables S1 and S2) show a highly significant difference between bower and control patterns (bower or court, indicated by BorC or BwOrCt in Supplementary Table S2), and also significant effects of angle and distance. Individual effects of bower or control names are not significant because they are overwhelmed by the BorC variable. The general conclusion is that the avenue reduces the width and extent of the best location to vocalize (Fig. 3).

**Fig. 4.**
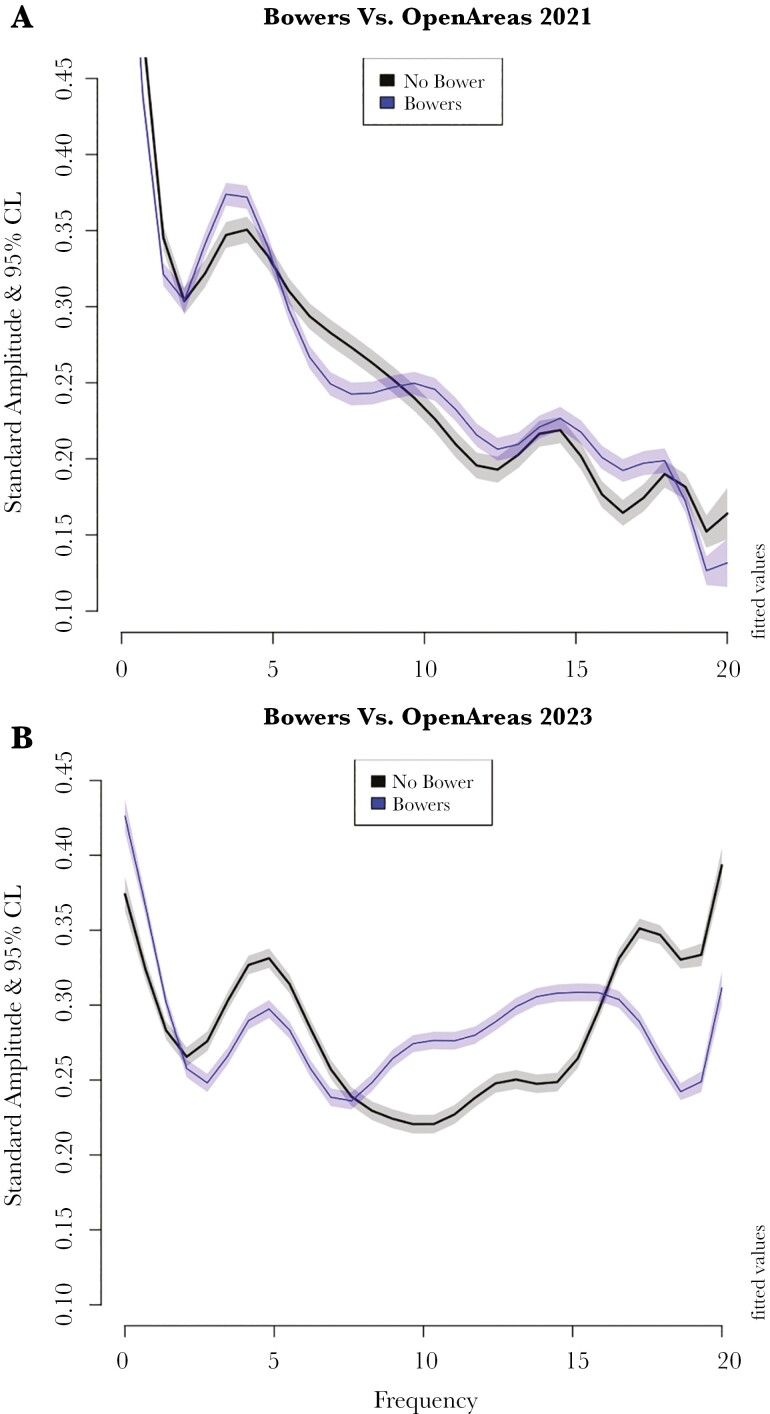
GAM Fitted Sound spectra (kHz) and their 95% CL arriving at the microphone positioned above the central depression with the bower present (blue), or the same geometry in an open area with no bower (black). Differences are significant when CL do not overlap. Each spectrum was adjusted to sum to 100 before the GAM analysis to avoid differences in intensity owing to among-bower geometry although bower was also included in the analysis. A) Directional microphone experiment (experiment 1) B) Omnidirectional microphone experiment (experiment 2) bowers enhance some sound frequencies and reduce others irrespective of sound equipment. The main reason for the difference between A and B is the speaker quality (see Supplementary Fig. S3). A colour version of this figure appears in the online version of this article.

In both experiments 1 and 2 the presence of the bower (court and avenue) significantly affects the sound spectrum arriving above the central depression in the avenue (Fig. 4). In both experiments a GAM (general additive model) shows highly significant (*P* < 10^−16^) interactions between sound frequency on bower vs no bower, angle and distance (analysis in Supplementary Table S2). The most interesting effect is the effect of the bower on the sound spectrum; affecting frequencies differently but consistently among the two experiments. Bowers were associated with increased relative (to position 1) Intensities over 8 to 16 kHz and reduced over 5 to 8 kHz relative to no bower, regardless of equipment (Fig. 4).

**Fig. 5.**
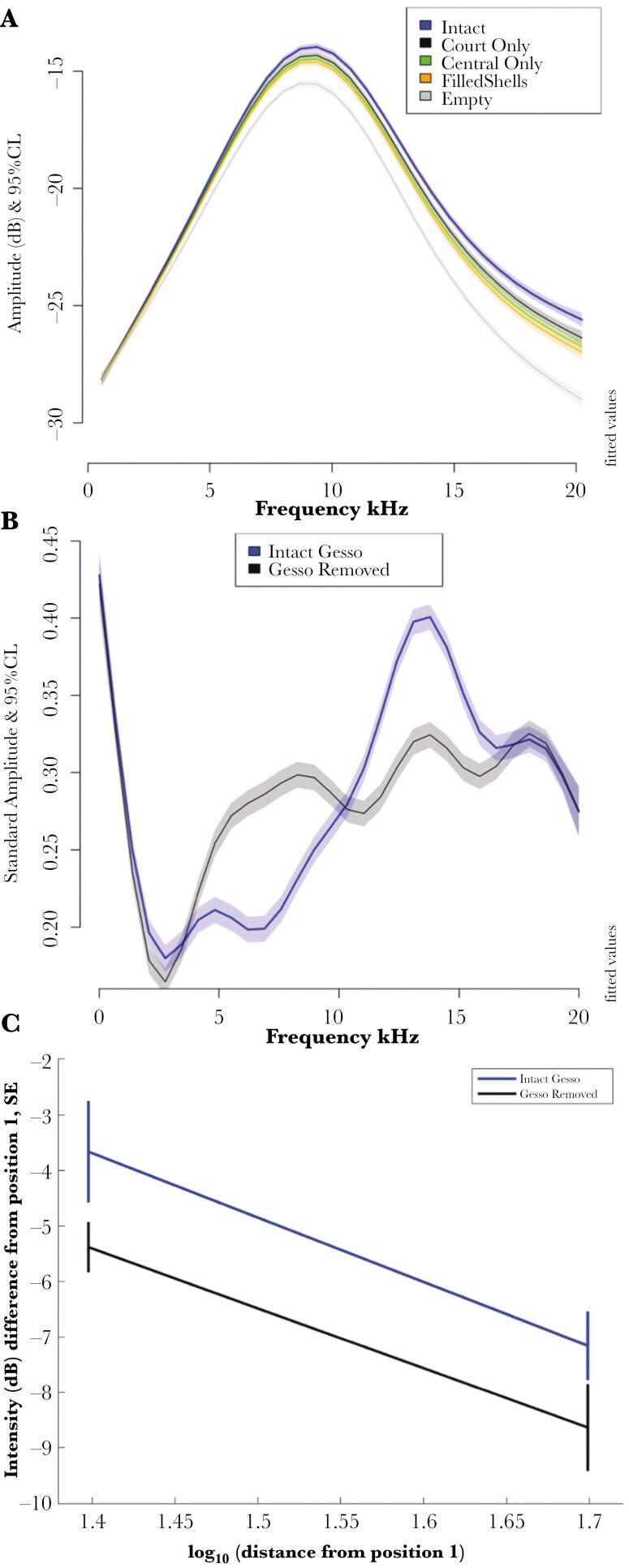
Gesso manipulation A) removal of gesso components (experiment 3). Vertical axis is dB to show intensity as well as spectral effects. As in Fig. 4, the shaded bands are 95% CL from the GAM. Intact: undisturbed bower. Court only: removing everything but the court. Central only: removing everything but objects in central depression. Filled shells: shells filled with clay. Empty: everything removed. B) Complete removal of all gesso (experiment 4); vertical axis as in Fig. 4. C) Total intensity at 2 positions along avenue axis before and after gesso removal (experiment 4). Note how gesso increases the total intensity and amplitude of higher frequencies in both experiments.

In order to explore the explicit effect of the gesso on the sound received at the female’s head position we performed two experiments, one removing specific gesso components (experiment 3) and the other removing the entire court gesso (experiment 4), see Fig. 5, analysis in Supplementary Tables S3 and S4. In both experiments, we recorded from the 3 positions on the avenue axis (to the left of position 1 in Fig. 1E). In both experiments removing gesso significantly reduced the relative intensity of sound arriving at the female’s head position (Fig. 5A, C; Supplementary Tables S4 and S5).

Removing individual gesso components on those bowers, which had abundant snail shells, showed that the most important contribution to intensity are the shells on the court; when removed the intensity and bandwidth both significantly declined (Fig 5A). There were much smaller effects from removing the objects from the central depression or filling the shells with clay (Fig. 5A); court gesso appears to be more important than avenue gesso.

In both experiments, the bandwidth of the signal significantly decreased when the gesso was removed (Fig. 5A, B). With the broader band and more sensitive equipment in experiment 4 (Fig 5B, C) we found a similar pattern to what we found in the 2 geometry experiments (Fig. 4); the gesso increases intensity at about 11 to 16 kHz and decreases intensity at about 3 to 8 kHz (Fig. 5) compared to increasing 8 to 16 kHz and decreasing 5 to 8 kHz (Fig 4). The qualitative effects are very similar using 3 different sets of equipment with different bandwidths and sensitivity (Figs. 4 and 5). The main effect of the Gesso objects on the court is to increase the bandwidth of sound arriving at the female while she watches the male’s display in the avenue as measured by the microphone in the female’s head position.

## Discussion

The shape of the bower avenue (Fig. 1) funnels the sound and results in a male’s vocalizations being loudest to the female in the avenue if he is at or close to the avenue entrance and also close to the avenue long axis (Fig. 3). This is exactly where he does most of his display sequence (see online video). He is closer to the avenue entrance because sound intensity decreases with distance due to physics (Dusenbery 1992), even over the short distances between the male and the female in the avenue; closeness matters. Female cowbirds actually respond to sounds with less intensity at greater distances (King et al. 1981) but this might not occur in Great Bowerbirds because they vocalize as close to the female as possible. During his display he moves his head across avenue entrance, consequently, the sound intensity reaching the female will vary during the display beyond that due to his own behavioural modulation and changing head direction (Patricelli et al. 2007, 2008; Great Bowerbird heads face away from the female during nuchal crest display, see online video). Most of his vocalisations during his display to a female consist of a continuous “tic-tic-tic” sound (vertical line pattern in Fig. 2A, expanded in Supplementary Fig. S1), alternating with “hisses” (vertical rectangles in Fig. 2A). Tic series are 5 to 10 s in duration and hisses are 1 to 1.5 s, and the tic series are performed as he moves his head across avenue entrance (see video). Although sound radiates from a source in all directions (Dusenbery 1992) there will be some position-independent modulation. When displaying an object in his bill, the male’s beak often points perpendicular to the avenue axis and when he displays his nuchal crest his bill faces away from the avenue entrance. Sound does not radiate from a bird’s head as a sphere but has a directional component allowing direction-dependent modulation as the head moves (Patricelli et al. 2007, 2008). Consequently, the amplitude of his vocalisations will vary during the display, even though almost all of his time is spent in the zone where his sound reaches the female at relatively highest amplitude (Fig. 3). We could not record this directly because males do not tolerate a microphone in their avenues when a female is present; they remove and destroy any unfamiliar objects placed in their avenues within a few minutes.

The statistical tests of geometry (Supplementary Table S2) show significant differences between the sound fields (Fig. 3) of bowers and controls (open area). There are also significant effects of distance and angle. The angle result is primarily due to the lateral restriction of sound by the tunnel-like avenue. The distance result is not surprising because sound declines with the reciprocal of the square root of distance. The lack of a site (bower or control) effect is mainly because variation among sites is much smaller than between bowers and controls.

The gesso (stones, bones, empty bleached snail shells) significantly intensifies and also broadens the sound spectrum arriving at the female’s head position as shown by comparisons between intact bowers and controls and between data recorded before and after the gesso is manipulated (Figs. 4 and 5; Supplementary Tables S2, S3, and S4). Figure 4 and Supplementary Table S2 show the spectral differences between bowers and controls. In both directional and omnidirectional experiments the courts in bowers deliver more higher frequency and fewer lower frequencies; increased over about 8 to 16 kHz and reduced over about 5 to 8 kHz relative to no bower, regardless of equipment. Gesso manipulation effects (Fig. 5; Supplementary Tables S3 and S4) are qualitatively similar, gesso presence is associated with intensity increases at about 11 to 16 kHz and decreases at about 3 to 8 kHz relative to the same bower with the gesso removed. These results are not surprising because the gesso objects are harder than the ground, twigs, detritus, and colored objects, hence are more reflective and also reflect more strongly at higher frequencies (Dusenbery 1992). For example, flat stones, which are ubiquitous in Great Bowerbird bowers, have a fairly flat reflection spectrum to at least 15 kHz (Isele et al. 2009).

Gesso is found on the court and often also in the avenue central depression (Fig. 1A). Although experiment 3 equipment had narrower band reception than the other experiments, it still showed that removing all gesso reduced the relative sound intensity and narrowed the spectrum arriving at the female’s position (Fig. 5A). This experiment also found the effects of court gesso much larger than that in the central depression. There are 2 possible explanations. First, the surface area of the court gesso is many times larger than the depression gesso, so would reflect more sound. Second, the internal shape of the avenue around the depression is complex, and there is a possibility that it may focus the sound towards the female as does the similarly-shaped but smaller mole cricket burrow (Daws et al. 1996). We did not try to estimate the focus because it would require complex and precise 3D manipulation of the microphone. Moreover, the avenue entrances are shaped as flaring horns, which are known to affect the sound in Mole crickets (Hill et al. 2006). The entrances and the internal enlargement over the central depression are reminiscent of the horn and bulb of Mole Crickets which significantly amplifies male sounds coming from his burrow (Daws et al. 1996; Bailey et al. 2001; Hill et al. 2006). In all other taxa using structures to augment auditory signals, males call in the structure. However, in Bowerbirds the male calls outside the avenue and the female sits within the avenue. In Bowerbirds the sound direction is reversed, from outside to inside. Here the avenue walls could focus the sound from the male to the female’s head. Since the avenue is much larger than a mole cricket burrow, if it does focus it would focus lower frequencies than in Mole crickets. Alternatively, the gesso in the central depression may reduce the focusing power but just increase the reflection from the avenue floor to the female. In any case, anything which captures and retains the female’s attention will be an advantage in mating. This focus conjecture needs further careful research.

Although the sound intensity and bandwidth enhancement of the ornaments are significant, we do not know what higher frequencies Bowerbirds can actually hear. Passerines can generally hear up to about 10 kHz and sensitivity declines rapidly above that even if they emit higher frequency sounds (Köppl 2022). Gleich (Gleich et al. 2005; Gleich and Langemann 2011) showed that there is a good relationship between body size, basilar papilla length, and the optimum and maximum audible sound frequency for all birds and provided equations. These equations seriously underestimate the maximum for hummingbirds yielding 8.5 kHz instead of the observed 15 kHz for *Oreotrochilus chomborazo* (Duque et al. 2020), and are based upon a tiny fraction of avian diversity. The equations for a bird with a Great Bowerbird mass (200 g) is predicted to be about 6 kHz, but given the very broad bandwidth of their tick, with almost equal intensity up to 15 kHz or even 20 kHz (Fig. 2; Supplementary Fig. S1 and Okida et al. 2010), this may also be an underestimate. Moreover, the spectral patterns do show differences in the possible hearing range of 6 to 1 kHz (Figs. 4 and 5). Why do they produce such broadband vocalizations unless they can hear them and make mating decisions based upon them? Direct spectral sensitivity measurements are needed, as in Duque et al. (2020).

Borgia (1995) suggested that the original function of the bower avenue is to provide some protection of a female inside the avenue against males displaying and running towards her. He was working with Satin Bowerbirds where the female does race towards the female, but this never happens in Great Bowerbirds. His was an argument about visibility within the avenue and “protection” of the female from an aggressive male. This was an argument about visibility within the avenue, but now we have an alternative and supplemental hypothesis, both visual and auditory. The avenue produces significant visual effects which capture and retain the female’s attention during a male’s display (Endler et al. 2014), and the auditory effects of the bower could also attract and retain a female’s attention, especially because, as the male moves his head in and out of her field of view, the amplitude of his display will go up and down (Fig. 3); habituation is less likely with a variable signal (Rankin et al. 2009). Protection against male aggression is not necessary since it does not occur in Great Bowerbirds and pure visibility and auditory effects are sufficient to explain the avenue function although that hypothesis is also relevant for Satin Bowerbirds.

Katsuno et al. (2010) found increasing mating success with longer and thicker-walled Great Bowerbird bower avenues. Longer avenues restrict the sound laterally more than short avenues and thicker walls also restrict sound coming from the sides with similar effects for visual signals. In fact, this is the basis for non-parabolic directional microphones. Given the continuous vocalizations during the male’s visual display, and that Satin Bowerbird female mating is affected by male vocalizations (Coleman et al. 2007), it is likely that females attend to both visual and auditory parts of the display; the bower is an efficient multimodal signal (Mitoyen et al. 2019).

The evolutionary sequence of sound and visual displays may be similar; they could have evolved simultaneously because they both could attract and hold the female’s attention. The evolutionary sequence for the avenue-building clade (Frith and Frith 2004; Endler et al. 2005) is: catbirds → toothbills → Regent (*Sericulus*) → Satin (*Ptilonorhynchus sensu strictu*) → Great, Spotted, Western (*Chlamydera* species, now lumped with *Ptilonorhynchus*). Endler et al. (2005) showed how starting with Regent Bowerbirds, bowers evolved longer and thicker avenues, larger areas of gesso, more hard gesso, and colors transferred from the plumage to the bowers. The visual changes could easily have resulted in a parallel evolutionary increase in the auditory properties as avenues became longer and thicker-walled, hence increasing in both visual and auditory directionality. For example, the avenues earlier in the phylogeny are much shorter, thinner, and have more soft court objects (Regent, Satin) and even the slightly later Spotted and Western Bowerbirds have thinner walls than Great and Fawn-Breasted Bowerbirds. Sound may have come first but its relative importance declined with evolutionary sequence as visual aspects of displays became more important. The most primitive Bowerbirds, catbirds and toothbilled Bowerbirds, have a large repertoire including some mimicry (catbirds do not have a bower, and toothbills only have a layer of leaves with white surfaces up leading to high visual contrast Frith and Frith 2004). Probably sound came first (catbirds), then in toothbills the visible display site added vision, and sound may have become less important as bowers became more complex and bower colors increased (Endler et al. 2005). But in all species sexual signaling is multimodal.

The maypole clade (Frith and Frith 2004) bowers are less likely to have directional sound properties since females are in a more open area covered with leaf litter or twigs and the bowers are mostly sticks, which do not reflect much in higher frequencies compared to hard objects. The Vogelkop Bowerbird makes a large hut-like structure with fruits outside and partially inside the structure. The male vocalizes close to the female inside the hut (Frith, pers. Comm. 2023), but the lack of hard structures (which strongly reflect sound) suggests that many sounds are absorbed particularly higher frequencies. This could be a way of reducing background noise and making the auditory display clearer. More work needs to be done on the auditory properties of Bowerbird bowers.

We have shown that Great Bowerbird bowers have complex interacting visual components (Endler et al. 2014) and that the same properties which create the visual effects also have auditory effects. The gesso provides a contrasting background for colored objects, and the nuchal crest and can induce visual illusions (Endler et al. 2014), but it also increases the intensity and bandwidth of the vocalisations. The avenue wall restricts the female’s field of view and provides chromatic adaptation but also restricts the male calling positions to produce maximum sound intensities only in the appropriate male display positions. There is also a chemical signal because males paint the inside avenue walls with saliva and plant material and females nibble the material whilst watching the male (Endler et al. 2014). The bower may therefore produce multimodal signals (Mitoyen et al. 2019), with the same structure producing visual, auditory, and chemosensory effects simultaneously. During avenue-building Bowerbird evolution the size and thatch density of bowers increased, (Endler et al. 2005) with Great Bowerbirds having the largest and longest avenues (Frith and Frith 2004). Based upon physics this implies that the auditory effects of the avenue also increased. But we do not know if one affected the evolution of the other or evolution was simultaneous.

At present, we have little direct evidence that female Great Bowerbirds make use of the sounds produced during the visual displays. Sounds are important in Satin Bowerbird courtship (Coleman et al. 2007) which have the second smallest avenues of avenue-building Bowerbird species and their avenue structure may not be solid enough for the effects we found in Great Bowerbirds. One problem is that the sound effects we measure could just be incidental to producing the visual effects. Moreover, sexual selection may favor the mode with more information (Ronald et al. 2012). However, some but not all species, multimodal signals are more effective than unimodal signals (Mitoyen et al. 2019). Finally, females and rival males could attend to the sounds as well as the visual displays. This needs direct experimentation.

Constructed devices to intensify and modify sound signals are also known in Mole crickets (Daws et al. 1996), Tree crickets (Mhatre et al. 2017), frogs (Muñoz and Halfwerk 2022), and fish (Kéver et al. 2014) but these are not multimodal, and, except for tree crickets, are burrows rather than constructions. Unlike the Bowerbirds these are all examples of structure augmenting signal transmission from the male whereas in Bowerbirds the construction affects what females receive; reception rather than transmission is augmented by the bower. Hummingbirds, like Bowerbirds, use physics to synchronize visual and vocal signals, taking advantage of the significantly directional gorget colors which flash during the motion and auditory display (Hogan and Stoddard 2018; Duque et al. 2022), in this case, the gorget flash is passive and the sounds and flight patterns are active. These display movements are likely to be energetically very costly. Having a physics-based flash saves extra energy which might otherwise be needed for active motion. What is particularly unusual about the Great Bowerbird multimodal display is that it is actively constructed by a male and affects the female reception of both visual and auditory signal components. No extra energy or neural circuitry is required for simultaneous multimodality in Great Bowerbirds; it is based upon the geometry and physical properties of the bower. Bowers take energy and effort to construct and maintain, but incidental effects of structure on sound require no additional energy. Energy is required for courtship movements but is the same for both visual and vocal displays, but additional energy is required for the vocalisations themselves. This suggests that any active use of received sound by females, if present, possibly evolved after the visual effects. Of course, the male also voluntarily uses display motion (see online video) and sound modulation (Fig. 2A) simultaneously as in other birds, but this is enhanced by the bower. There is presently no way to tell whether the visual or vocal aspects of Bowerbird displays came first and they could have evolved simultaneously, just as they are displayed simultaneously. The causes of evolution of multimodal displays needs more investigation.

These results lead to 4 general conclusions. (1) As for visual signals in Bowerbirds, animals can use structures to enhance and modulate auditory display, reducing the per display energetic costs. The physical separation from the structure and the signaler may also reduce predation risk (Endler et al. 2005) for both the visual and auditory parts of a signal. (2) It is important to consider the geometry of signaling structures and the position of the signaler and receiver (Echeverri et al. 2021), again for both visual and vocal displays. (3) Multimodal signaling can occur simultaneously if the structure affects visibility and sound, especially in animal-built structures. (4) We need to understand how multimodal signaling evolved given that it is widespread.

## Supplementary Material

arae070_suppl_Supplementary_Materials

arae070_suppl_Supplementary_Data

## Data Availability

Data analyzed in this article, along with a sample R script for the GAM analyses are found in DRYAD, doi:10.5061/dryad.6djh9w19w.
