# Supplementary material for: Acoustic effects complement visual displays of Great Bowerbird bowers
Source: Behav Ecol. 2024 Sep 7;35(6):arae070. doi: 10.1093/beheco/arae070 (PMC11486916; doi:10.1093/beheco/arae070)
Supplement: arae070_suppl_Supplementary_Materials [file arae070_suppl_supplementary_materials.pdf]

Online Supplement to

Acoustic effects of Great Bowerbird Bowers

John A. Endler, Selina Meehan, Aida Rodrigues, and Vicki Hallett

Contents:

Figure S1, Components of display vocalizations

Figure S2, Assessment of omnidirectionality of Marantz speaker

Figure S3, Comparison of microphones and speakers

Table S1, R scripts for GAM and GLM

Table S2, Results of GAM on spectra for geometry experiments

Table S3, Results of gesso component removal (2015)

Table S4, Results of complete gesso removal (2023)

Figure S1, Sound properties of Great Bowerbird vocal display components

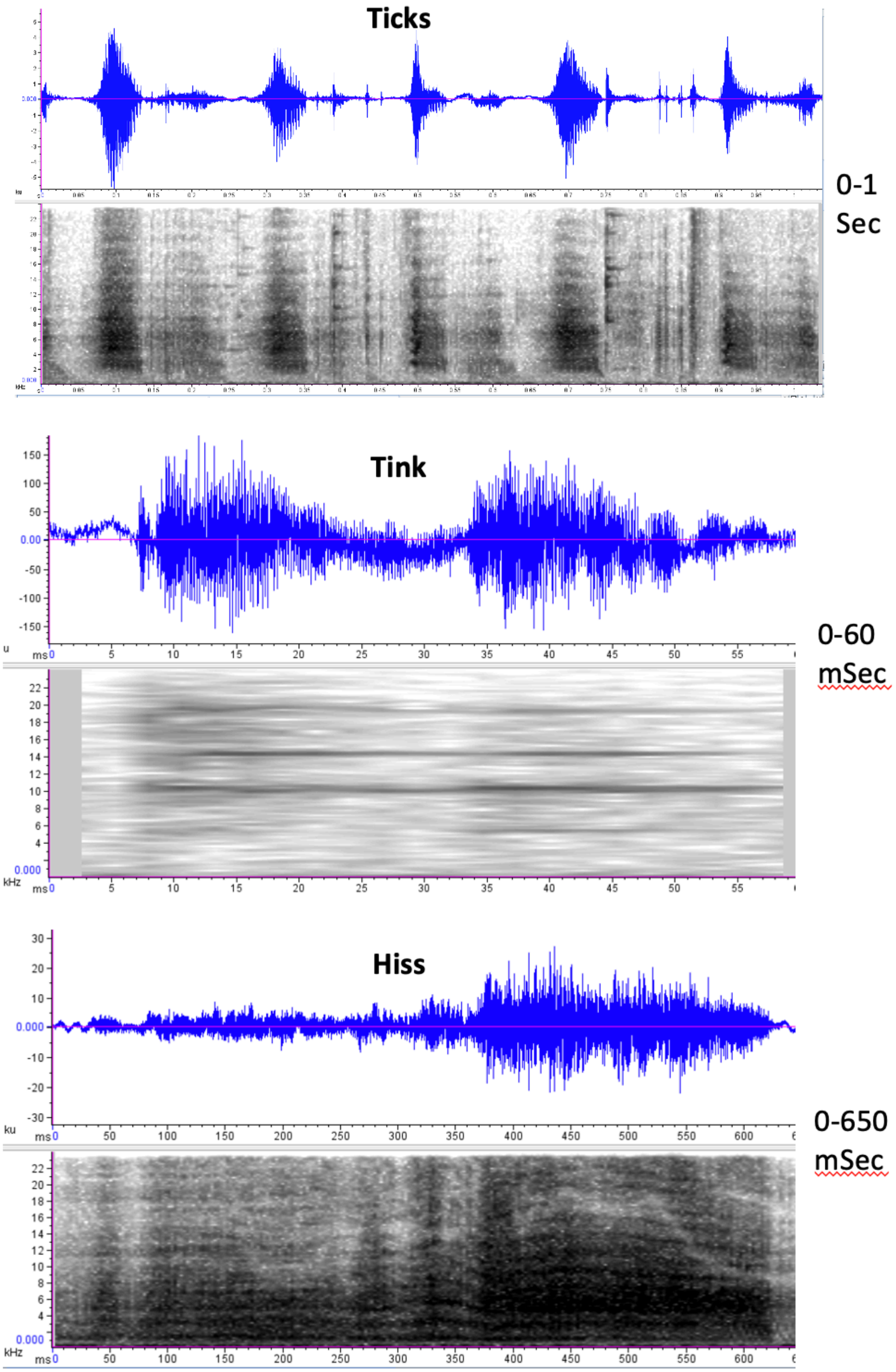

Figure S2, Effective omnidirectionality of the Marantz speaker.

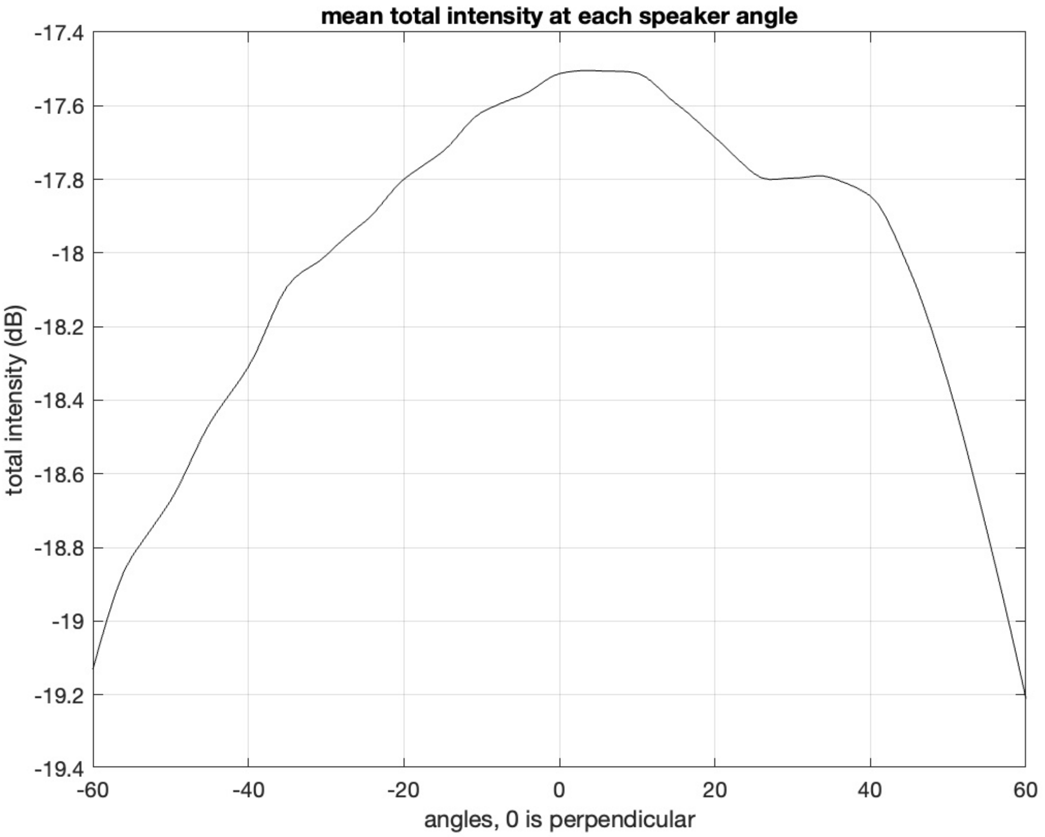

Figure S2. Directionality measurement of the Marantz PMD661 recorder speaker. We set the AudioTechnica AT8035 directional microphone facing the centre of a turntable in an open area. The turntable was marked in 10° intervals and was 1.5m from the microphone tip. The Marantz recorder played back the flat test spectrum sound (Fig. 2 I) from each angle to the tip of the directional microphone from -60° to 60° every 10° recorded by a second Marantz PMD660 recorder. The total intensities for each angle are shown in the figure.

Figure S3, Speaker Comparison

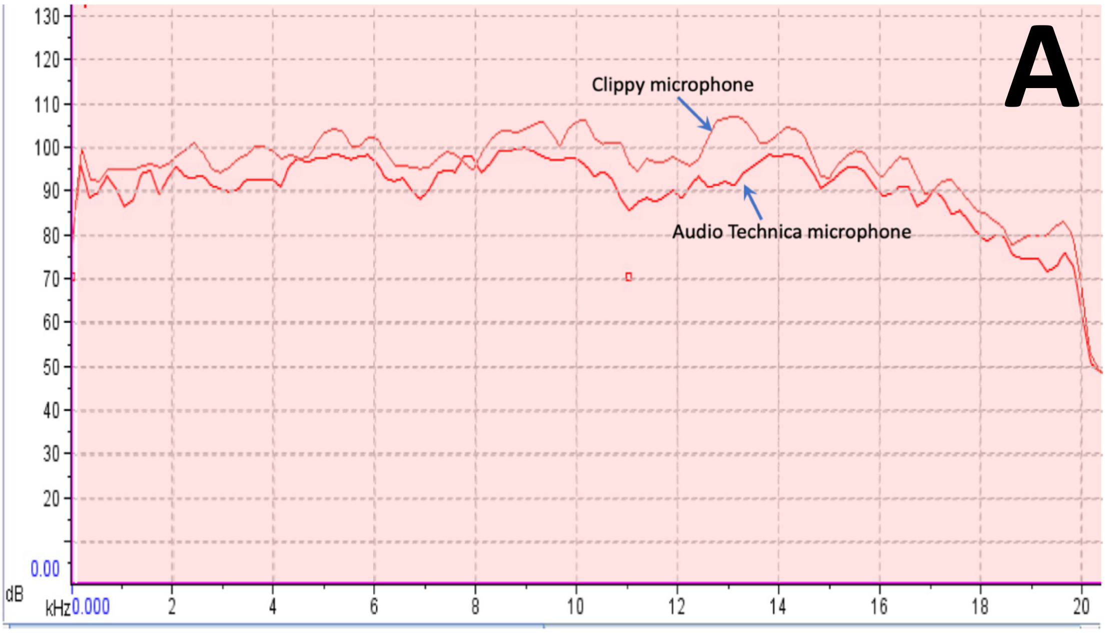

Both microphones tested with the same Marantz recorder and Bose Revolve
Speaker playing the standard frequency sweep at 1.3m from the microphone tip

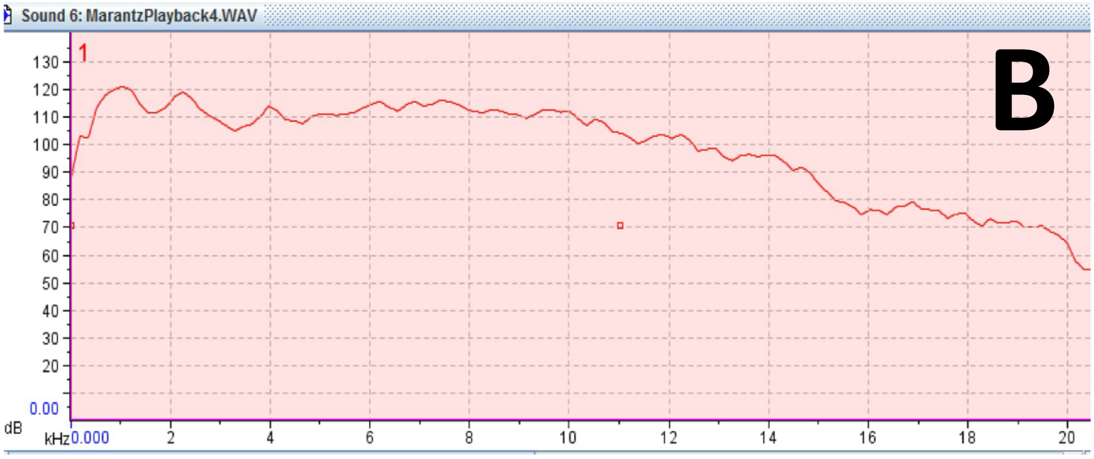

Marantz speaker playback to Clippy microphone.
The higher frequencies are attenuated relative to the Bose Revolve speaker.

Figure S3. Comparison of both microphones (2021, 2023) receiving sound from the Bose Surround speaker (A), or the Clippy microphone from the Marantz speaker (B). Analysis using RavenPro 1.4. Note how the two microphones are not very different (A) but the Marantz speaker (B) is poorer in emitting higher frequencies than the Bose Surround (A).

Table S1, R scripts used in GAM and GLM analyses

Setup for all GAM analyses in R

```
library(nlme)
library(mgcv)
library(itsadug)     #for plot_smooth
dta<-read.csv('<file_name.csv')   #file name changes for each analysis
dta$Site<-as.factor(dta$Site)
dta$BorC<-as.factor(dta$BorC)
dta$Angle<-abs(dta$Angle)     #ignore angle sign
md<-
gam(Amplitude~s(Frequency)+s(Frequency,BorC,bs="fs",m=1)+s(Frequency,Angle,bs="fs",m
=1)+s(Frequency,Distance,bs="fs",m=1)+s(Site,bs="re"),data=dta,method="REML")
summary(md)
dev.new()
pB<-plot_smooth(m4, view = "Frequency", rm.ranef = FALSE,
        cond = list(BorC="C"),
        main = "Bowers Vs. OpenAreas 2021", col = 'black', lwd=2,
        ylab='Standard Amplitude & 95% CL',ylim=c(0.1,0.45))
pC<-plot_smooth(m4, view = "Frequency", rm.ranef = FALSE,
        cond = list(BorC="B"),
        add = TRUE, col = 'blue')
legend("topright",legend=c("No Bower","Bowers"),fill=(col=c("black","blue")))
```

Setup for GLM analysis in R

Gesso Component Removal (2015)

```
library(lme4)
library(lmerTest)
dta=read.csv("ObjectExIntensityDataRaw.csv")
dta$Bower<-as.factor(dta$Bower)
dta$Exper<-as.factor(dta$Exper)
dta$Distance<-as.double(dta$Distance)
md<-lmer(TotCPos0~Distance+Exper+(1|Bower),dat=dta)
summary(md)
```

Complete Gesso Removal (2023)

```
library(nlme)
library(mgcv)
dta<-read.csv('BeforeAfterData.csv')   #repeat analysis with 60cm
dta$Bower<-as.factor(dta$Bower)
dta$BfAf<-as.factor(dta$BfAf)
mod<-lmer(Intensity~Distance+BfAf+(1|Bower),data=data)
summary(mod)
```

Table S2, Results of GAM on spectra from Directional Microphone and Omnidirectional Geometry data 2021 and 2023 (Figures 4 and 5)

Directional (2021):

```
Formula:
Amplitude ~ s(Frequency) + s(Frequency, BorC, bs = "fs", m = 1) +
    s(Frequency, Angle, bs = "fs", m = 1) + s(Frequency, Distance,
    bs = "fs", m = 1) + s(Site, bs = "re")

Parametric coefficients:
             Estimate Std. Error t value Pr(>|t|)
(Intercept) 0.2597403  0.0006024   431.2   <2e-16 ***
---
Signif. codes:  0 '***' 0.001 '**' 0.01 '*' 0.05 '.' 0.1 ' ' 1
Approximate significance of smooth terms:
                             edf Ref.df     F p-value
s(Frequency)           8.274e+00  8.335 91.24  <2e-16 ***
s(Frequency,BorC)      1.260e+01 17.000 44.04  <2e-16 ***
s(Frequency,Angle)     1.959e+01 19.979 42.82  <2e-16 ***
s(Frequency,Distance) 1.924e+01 19.915 26.79  <2e-16 ***
s(Site)                7.113e-04  7.000  0.00       1
---
Signif. codes:  0 '***' 0.001 '**' 0.01 '*' 0.05 '.' 0.1 ' ' 1
---
R-sq.(adj) =  0.396   Deviance explained = 39.7%
-REML = -24332  Scale est. = 0.010182  n = 28105
```

Omnidirectional (2023):

```
Formula:
Amplitude ~ s(Frequency) + s(Frequency, BwOrCt, bs = "fs", m = 1) +
    s(Frequency, Angle, bs = "fs", m = 1) + s(Frequency, Distance,
    bs = "fs", m = 1) + s(Bower, bs = "re")

Parametric coefficients:
             Estimate Std. Error t value Pr(>|t|)
(Intercept) 0.2824856  0.0004503   627.3   <2e-16 ***
---
Signif. codes:  0 '***' 0.001 '**' 0.01 '*' 0.05 '.' 0.1 ' ' 1
Approximate significance of smooth terms:
                            edf Ref.df      F  p-value
s(Frequency)            1.00008   1.00  21.46 3.59e-06 ***
s(Frequency,BwOrCt)    14.07731  17.00 179.20  < 2e-16 ***
s(Frequency,Angle)     23.77994  23.94  60.84  < 2e-16 ***
s(Frequency,Distance) 17.86045  19.49  16.06  < 2e-16 ***
s(Bower)                0.01613  15.00   0.00        1
---
Signif. codes:  0 '***' 0.001 '**' 0.01 '*' 0.05 '.' 0.1 ' ' 1
---
R-sq.(adj) =   0.12   Deviance explained = 12.1%
-REML = -49006  Scale est. = 0.013553  n = 67260
```

Table S3, Results of Gesso Component Removal (2015)

Gesso Component Removal GLM on total intensity

```
Linear mixed model fit by REML. t-tests use Satterthwaite's method
['lmerModLmerTest']
Formula: TotIntensity ~ Distance + Exper + (1 | Bower)
   Data: dta
REML criterion at convergence: 111.1
Scaled residuals: 
    Min      1Q  Median      3Q     Max 
-1.8998 -0.5083  0.1934  0.6716  1.6674 
Random effects:
 Groups   Name        Variance Std.Dev.
 Bower    (Intercept) 0.1792   0.4233  
 Residual             0.2932   0.5415  
Number of obs: 57, groups:  Bower, 4
Fixed effects:
             Estimate Std. Error        df t value Pr(>|t|)    
(Intercept) -0.457594   0.277381  6.923845  -1.650   0.1435    
Distance    -0.065438   0.003513 47.984826 -18.625  < 2e-16 ***
ExperCD0    -0.452067   0.221051 47.984826  -2.045   0.0464 *  
ExperCt0    -0.358783   0.221051 47.984826  -1.623   0.1111    
ExperFSS    -0.581118   0.242486 48.368475  -2.397   0.0205 *  
ExperWSS    -1.165900   0.221051 47.984826  -5.274 3.15e-06 ***
---
Signif. codes:  0 '***' 0.001 '**' 0.01 '*' 0.05 '.' 0.1 ' ' 1
Correlation of Fixed Effects:
         (Intr) Distnc ExpCD0 ExprC0 ExpFSS
Distance -0.317                            
ExperCD0 -0.398  0.000                     
ExperCt0 -0.398  0.000  0.500              
ExperFSS -0.363  0.000  0.456  0.456       
ExperWSS -0.398  0.000  0.500  0.500  0.456
```

Gesso Component Removal GAM

```
Family: gaussian 
Link function: identity 
Formula:
Amplitude ~ s(Frequency) + s(Frequency, Exper, bs = "re") + 
s(Frequency, 
    Distance, bs = "re")
Parametric coefficients:
            Estimate Std. Error t value Pr(>|t|)    
(Intercept)   -22.92       1.14  -20.11   <2e-16 ***
---
Signif. codes:  0 '***' 0.001 '**' 0.01 '*' 0.05 '.' 0.1 ' ' 1
Approximate significance of smooth terms:
                        edf Ref.df      F p-value    
s(Frequency)          8.791  8.987 3749.0  <2e-16 ***
s(Frequency,Exper)    3.981  4.000  223.9  <2e-16 ***
s(Frequency,Distance) 1.999  2.000 3006.6  <2e-16 ***
---
Signif. codes:  0 '***' 0.001 '**' 0.01 '*' 0.05 '.' 0.1 ' ' 1
R-sq.(adj) =  0.928   Deviance explained = 92.8%
-REML = 6414.1  Scale est. = 1.9249    n = 3648
```

Table S4, Results of Complete Gesso (Court) Removal, 2023

Complete Gesso Removal (Present then absent) GLM

```
Linear mixed model fit by REML. t-tests use Satterthwaite's method
['lmerModLmerTest']
Formula: Intensity ~ Distance + BfAf + (1 | Bower)
   Data: data
REML criterion at convergence: 169.2
Scaled residuals: 
     Min       1Q   Median       3Q      Max 
-1.63637 -0.69003  0.04984  0.53528  2.31174 
Random effects:
 Groups   Name        Variance Std.Dev.
 Bower    (Intercept) 2.457    1.568   
 Residual             2.571    1.603   
Number of obs: 40, groups:  Bower, 10
Fixed effects:
            Estimate Std. Error      df t value Pr(>|t|)    
(Intercept)  -1.9550     0.9760 35.6915  -2.003  0.05281 .  
Distance     -0.1123     0.0169 28.0000  -6.647 3.27e-07 ***
BfAfB         1.6000     0.5070 28.0000   3.156  0.00381 ** 
---
Signif. codes:  0 ‘***’ 0.001 ‘**’ 0.01 ‘*’ 0.05 ‘.’ 0.1 ‘ ’ 1
---
Correlation of Fixed Effects:
         (Intr) Distnc
Distance -0.779       
BfAfB    -0.260  0.000
```

Complete Gesso Removal (Present then absent) GAM

```
Family: gaussian 
Link function: identity 
Formula:
Amplitude ~ s(Frequency) + s(Frequency, BfAf, bs = "fs", m = 1) + 
    s(Bower, bs = "re") + s(Distance, bs = "re") + s(Frequency, 
    Distance, bs = "fs", m = 1)
Parametric coefficients:
            Estimate Std. Error t value Pr(>|t|)    
(Intercept) 0.282485   0.001448   195.1   <2e-16 ***
---
Signif. codes:  0 ‘***’ 0.001 ‘**’ 0.01 ‘*’ 0.05 ‘.’ 0.1 ‘ ’ 1
---
Approximate significance of smooth terms:
                             edf Ref.df      F p-value    
s(Frequency)           6.893e+00  6.971  2.334  0.0361 *  
s(Frequency,BfAf)      8.371e+00 17.000 29.455  <2e-16 ***
s(Bower)               6.853e-04  9.000  0.000  1.0000    
s(Distance)            8.909e-28  1.000  0.000  0.4668    
s(Frequency,Distance) 1.967e+01 21.225  9.341  <2e-16 ***
---
Signif. codes:  0 ‘***’ 0.001 ‘**’ 0.01 ‘*’ 0.05 ‘.’ 0.1 ‘ ’ 1
---
R-sq.(adj) =  0.194   Deviance explained = 19.6%
-REML = -9270.8  Scale est. = 0.015613  n = 14160
```

Video of a typical Great Bowerbird Display
(separate file)
